# An in vitro method to evaluate hemolysis of human red blood cells (RBCs) treated by airborne particulate matter (PM_10_)

**DOI:** 10.1016/j.mex.2019.01.001

**Published:** 2019-01-10

**Authors:** Alireza Mesdaghinia, Zahra Pourpak, Kazem Naddafi, Ramin Nabizadeh Nodehi, Zahra Alizadeh, Soheila Rezaei, Amir Mohammadi, Maryam Faraji

**Affiliations:** a Department of Environmental Health Engineering, School of Public Health, Tehran University of Medical Sciences, Tehran, Iran; b Center for Air Pollution Research (CAPR), Institute for Environmental Research (IER), Tehran University of Medical Sciences, Tehran, Iran; c Immunology, Asthma and Allergy Research Institute, Tehran University of Medical Sciences, Tehran, Iran; d Social Determinants of Health Research Center, Yasuj University of Medical Sciences, Yasuj, Iran; e Department of Public Health, School of Nursing and Midwifery, Maragheh University of Medical Sciences, Maragheh, Iran; f Environmental Health Engineering Research Center, Kerman University of Medical Sciences, Kerman, Iran; g Department of Environmental Health, School of Public Health, Kerman University of Medical Sciences, Kerman, Iran

**Keywords:** Effect of airborne particulate matter on hemolysis in vitro, Cyanmethemoglobin, Hemoglobine, Hemolysis percent, Air pollution, Dry extraction, In vitro test, ASTM standard E2524-08, Hemolytic samples

## Abstract

Air pollutants are capable to enter bloodstream through the nose, mouth, skin and the digestive tract. Hemolysis is the premature destruction of red blood cells (RBCs) membranes. This can affect metabolism of RBCs and reduce cell life. Each of these adverse effects could lead to anemia, jaundice and other pathological conditions. Hemolysis can induce by the mineral components adsorbed on the particles. The aim of this study was to evaluate hemolysis of RBCs treated by airborne PM_10_ (PM with aerodynamic diameter ≤ 10 μm) in vitro. Study had two main stages including sampling and preparation of PM_10_ suspension, and hemolysis test. Particle samples were collected by means of a high-volume sampler on fiberglass filters. The PM_10_ was extracted through dry ultrasonic method. Blood sample was incubated by PM_10_ at concentrations 50–300 μg/mL for 3 h. Hemolysis percent was assessed through measurement of Hemoglobin concentration in test samples and total blood hemoglobin (TBH) sample by the cyanmethemoglobin method. Analysis of variance (ANOVA) and Tukey post-hoc test were applied to compare mean values of hemolysis percent between different PM concentrations.

Method used in current study is suggested for investigation of toxic effects of airborne particle matter (PM_1_, PM_2.5_ and PM_10_) on human RBCs.

**Specifications Table**

**Table:** 

Subject Area	• Environmental Science
More specific subject area:	Hemolysis effect of air pollutants
Method name:	Effect of airborne particulate matter on hemolysis in vitro
Name and reference of original method	Method for Analysis of Nanoparticle Hemolytic Properties in Vitro https://doi.org/DOI:10.1021/nl0805615
Resource availability	–

## Method details

**Fig. 1:**
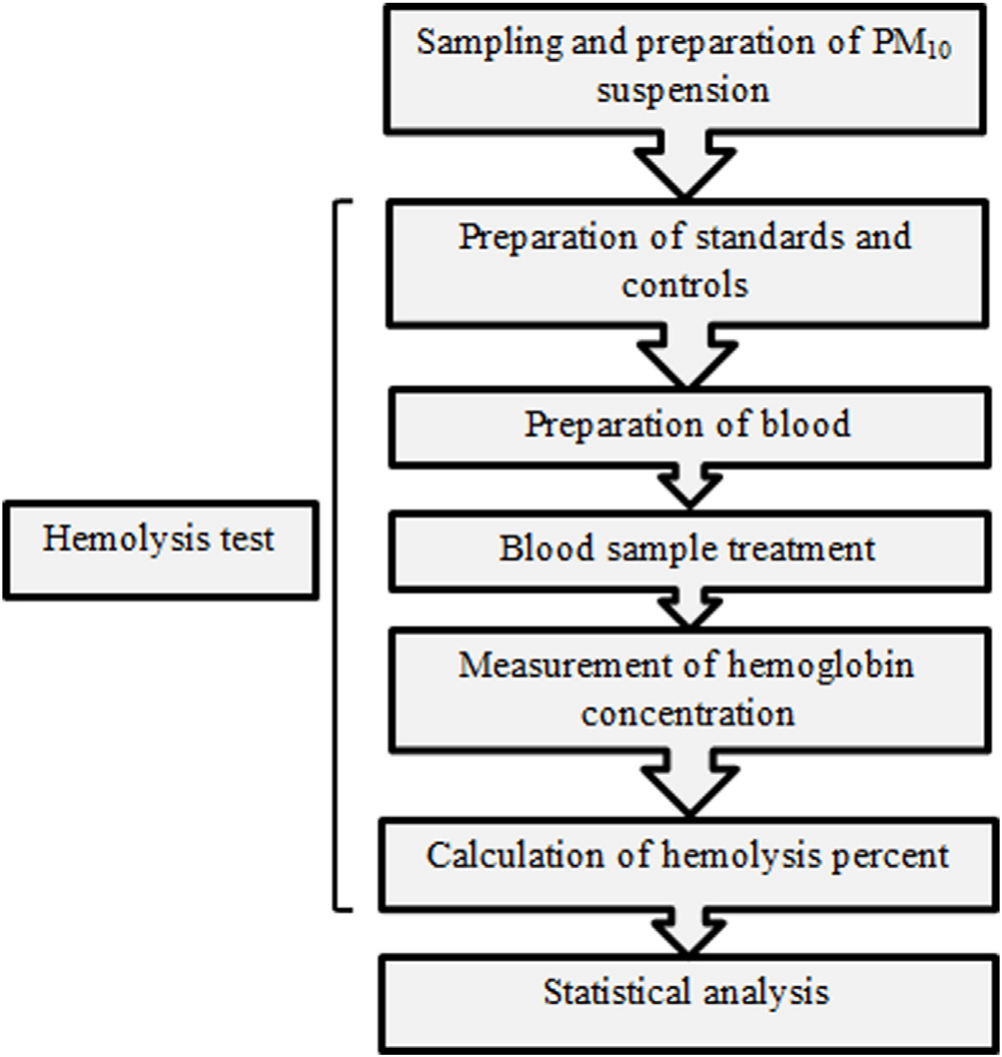
Flow diagram of the study stages.

Current study was done in the stages as follows: sampling and preparation of PM_10_ suspension and hemolysis test including preparation of standards and controls, preparation of blood, blood sample treatment, and measurement of hemoglobin concentration and calculation of hemolysis percent. Finally, statistical analysis was carried out on data. Flow diagram of the study stages are exhibited in Fig. 1.

## Sampling and preparation of PM_10_ suspension

Sampling of particle was described in detail in our previous works [1]. Briefly, PM_10_ was sampled by a high-volume sampler (1.3–1.7 m^3^/min) (Grasebey, USA) on the fiberglass filter (8 × 10 in., grade G 653 Whatman, USA). Sampled particles was extracted as the dry ultrasonic method that described in detail before [2]. PM_10_ suspension was prepared in phosphate buffered saline (PBS) (Biosera, France) at six concentrations of 50, 100, 150, 200, 250 and 300 μg/mL.

## Hemolysis test

Hemolysis test were surveyed in the basis of ASTM standard E2524-08 (Standard Test Method for Analysis of Hemolytic Properties of Nanoparticles) with some modifications in the concentration of analyzed particles [3,4]. The ethics committee of the Tehran University of Medical Sciences approved current study (IR.TUMS.SPH.REC.1395.841). Written informed consent was obtained from volunteers and their parents before initiating the study.

### Preparation of standards and controls

#### Standards

Hemoglobin standards were prepared by means of hemoglobin standard (StanBio Laboratory, Boerne, TX, USA) and cyanmethemoglobin (CMH) reagent (Sigma-Aldrich, St. Louis, MO, USA) that used to construct a standard curve at the range of hemoglobin concentration from 0 to 200 mg/mL (Table 1).

**Table 1:** Preparation of Calibration Standards.

Standard	Nominal Concentration (mg/mL)	Preparation Procedure	
		Standard (μL)	CMH reagent (μL)
S1	0	0	200
S2	80	80	120
S3	120	120	80
S4	160	160	40
S5	180	180	20
S6	200	200	0

#### Positive control

Triton X-100 (Sigma-Aldrich, St. Louis, MO, USA) at a stock concentration of 1% (10 mg/mL) was used as the assay positive control. Triton X-100 can be prepared in the sterile distilled water and kept at 4 °C for up to 2 weeks.

#### Negative control

Sterile solution of PBS was used as the negative control that could store the stock solution at room temperature.

#### Blood-free control

Particles that could not be removed after centrifugation of samples may cause false positive results. These false positive results were corrected by aliquots of PM_10_ suspension at corresponding concentrations those used in the assay in PBS without blood.

#### Inhibition/enhancement control (IEC)

This control is used to approximate potential interaction between air particles and hemoglobin released in the supernatant, which may mask hemoglobin from detection, by the assay. False negative results were corrected by spiking cell-free supernatant, from the positive control after centrifugation, to particles at corresponding concentrations those used in the assay [3,4].

### Preparation of blood

Whole blood from three donors 16–18 years old was collected into a syringe containing heparin as an anti-coagulant. The inclusion criteria for volunteers were: being healthy at the time of taking blood, nonsmoking (no smoking at least 6 months before the study), no taking regular medication, no experiencing any inflammation during blood sampling and one week ago, and no occupational exposure to environmental pollutants. Equal proportions of collected whole blood were pooled and diluted ten times with PBS. Diluted blood was used in all of the samples with blood.

### Blood sample treatment

Aliquots (100 μl) of PM_10_ suspension in PBS at the mentioned mass concentrations were added to microcentrifuge tubes. Then, 700 μl of PBS and 100 μl of diluted blood were added to the sample, positive and negative control tubes. All of samples were prepared in triplicate. These were incubated at a 37 °C water bath for 3 h, following gentle inversion of the tubes every 30 min. After the incubation, particles and intact RBCs were removed through centrifugation of tubes at 800 *g* for 15 min at room temperature.

### Measurement of hemoglobin concentration

Hemoglobin concentration was measured by the cyanmethemoglobin method [3,4]. Total blood hemoglobin (TBH) was prepared by combining 20 μL of diluted blood with 5 mL of CMH reagent.

**Fig. 2:**
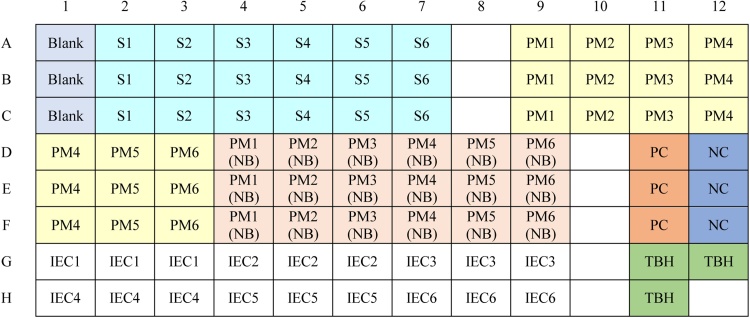
Plate map; S = Standard, PMn = Particle matter at concentrations of 1–6 corresponding to 50–300 μg/mL, NB = No blood, PC = Positive control, NC = Negative control, IECn = Inhibition/enhancement control at concentrations of 1–6 corresponding to 50–300 μg/mL, TBH = Total blood hemoglobin.

200 μL of blank (CMH reagent), total blood hemoglobin (TBH) sample that its preparation described above and standards (Table 1) was added in each well on a 96 well plate (See Plate map in Fig. 2). Also, 100 μL of supernatant of test samples and controls (positive, negative, blood-free and IEC) was added on the plate and mixed in a 1:1 ratio with cyanmethemoglobin (CMH) reagent. Absorbance of each sample was read at an absorbance wavelength of 540 nm. Hemoglobin concentration was calculated according to the standard curve constructed through hemoglobin standards. Dilution factor 18 was accounted for samples and controls, and 251 for TBH.

### Calculation of hemolysis percent

Hemolysis percent was calculated from Eq. (1) by dividing each sample’s hemoglobin concentration by the TBH.

(1) $$\text{Hemolysis}\;\;\left(\%\right)=\text{Hemoglobine}\;\;\text{in}\;\text{test}\;\text{sample/TBH}\times 100$$

Hemolysis percent in samples is interpreted as follow: <2%: non-hemolytic, 2–5%: slightly hemolytic and >5% hemolytic samples [3].

## Statistical analysis

The effect of PM_10_ concentration at six levels (50, 100, 150, 200, 250 and 300 μg/ml) on the hemolysis percent as the response was investigated with R software version 3.4.3 [5,6]. Significant level was considered as p-value less than 0.05. Analysis of variance (ANOVA) followed by Tukey post-hoc test was applied to compare the hemolysis percent between different PM concentrations.

## Conflict of interest

The authors declare that they have no competing financial interests.
